# Circulating MicroRNAs in Gastrointestinal Cancer

**DOI:** 10.3390/cancers13133348

**Published:** 2021-07-03

**Authors:** Masahisa Ohtsuka, Kazuya Iwamoto, Atsushi Naito, Mitsunobu Imasato, Satoshi Hyuga, Yujiro Nakahara, Manabu Mikamori, Kenta Furukawa, Jeongho Moon, Tadafumi Asaoka, Kentaro Kishi, Awad Shamma, Hiroki Akamatsu, Tsunekazu Mizushima, Hirofumi Yamamoto

**Affiliations:** 1 Department of Surgery, Osaka Police Hospital, 10-31 Kitayama-cho, Tennouji-ku, Osaka 543-0035, Japan; iwamoto.kazuya@khaki.plala.or.jp (K.I.); naito@oph.gr.jp (A.N.); imasato@oph.gr.jp (M.I.); satoshihyuuga0831@gmail.com (S.H.); yujiro0210@yahoo.co.jp (Y.N.); mmikamori@oph.gr.jp (M.M.); kfurukawa@oph.gr.jp (K.F.); c-mun@oph.gr.jp (J.M.); tasaoka@oph.gr.jp (T.A.); kishi-ke@oph.gr.jp (K.K.); h.akamatsu@me.com (H.A.); tmizushima@oph.gr.jp (T.M.); 2 Department of Molecular Pathology, Division of Health Sciences, Graduate School of Medicine, Osaka University, Yamadaoka 1-7, Suita, Osaka 565-0871, Japan; shamawad@sahs.med.osaka-u.ac.jp (A.S.); hyamamoto@sahs.med.osaka-u.ac.jp (H.Y.)

**Keywords:** circulating microRNA, gastrointestinal cancer, noninvasive tumor markers

## Abstract

**Simple Summary:**

The screening methods and therapeutic strategies for gastrointestinal cancer (GIC) have improved, but mortality in GIC patients remains high. Early detection and precise evaluation of GIC are required to further improve treatment outcomes in GIC patients. MicroRNAs (miRNAs), which do not encode proteins, have attracted attention as biomarkers of various diseases. Since the first report revealing the strong correlation between miRNAs and cancer in 2002, numerous studies have illustrated the changes in the expression and the biological and oncological effects of miRNAs in GIC. Furthermore, miRNAs circulating in the blood are reported to be associated with GIC status. These miRNAs are thought to be useful as noninvasive biomarkers because of their stability in blood. Herein, we discuss the potential of miRNAs as noninvasive biomarkers for each type of GIC on the basis of previous reports and describe perspectives for their future application.

**Abstract:**

Gastrointestinal cancer (GIC) is a common disease and is considered to be the leading cause of cancer-related death worldwide; thus, new diagnostic and therapeutic strategies for GIC are urgently required. Noncoding RNAs (ncRNAs) are functional RNAs that are transcribed from the genome but do not encode proteins. MicroRNAs (miRNAs) are short ncRNAs that are reported to function as both oncogenes and tumor suppressors. Moreover, several miRNA-based drugs are currently proceeding to clinical trials for various diseases, including cancer. In recent years, the stability of circulating miRNAs in blood has been demonstrated. This is of interest because these miRNAs could be potential noninvasive biomarkers of cancer. In this review, we focus on circulating miRNAs associated with GIC and discuss their potential as novel biomarkers.

## 1. Introduction

Cancer is the third leading cause of death globally, and almost 9.6 million cancer-related deaths worldwide were reported in 2018. Global epidemiological data show that, of the various cancers, gastrointestinal cancer (GIC) causes 3.4 million deaths annually, accounting for more than one-third of all cancer-related deaths and making it the leading cause of cancer-related death. Colorectal cancer (CRC) is the second leading cause (9.2%) of cancer-relate d deaths, followed by gastric cancer (8.2%), liver cancer (8.2%), and esophageal cancer (5.3%) [1]. Although current medical technology improves the clinical course of GIC, mortality in GIC patients remains high. In cases of complete surgical resection in the early stages of GIC, the resulting prognosis is often favorable. Chemotherapy, radiotherapy, and immunotherapy can improve advanced GIC, but the prognosis is generally poor. Therefore, new technology to detect early-stage GIC and therapeutic methods and targets are required.

Noncoding RNAs do not encode proteins but regulate their expression levels via post-transcriptional regulation. MicroRNAs (miRNAs) are short noncoding RNAs of 20–25 nucleotides that bind to the 3′ end of the untranslated region (3′UTR) of target genes. Since Calin et al. [2] revealed the relationship between human cancer and miRNAs, several reports have revealed miRNAs as onco-miRNAs or tumor suppressor miRNAs in human cancers. Because of their stability, miRNAs are thought to be ideal potential biomarkers of human cancer; however, there are several difficulties in miRNA-targeted therapy, including the delivery system. Moreover, currently, there are a limited number of ongoing clinical studies. Furthermore, circulating miRNAs, which are expressed in body fluid, can predict tumor progression, including lymph node metastasis, and aid in the prognosis and response to anticancer therapy in GIC patients [3,4,5,6,7,8].

In this review, we focus on circulating miRNAs in GIC and provide an overview of the role of circulating miRNAs, discussing their biological functions and their potential as tumor markers and therapeutic targets.

## 2. MicroRNA Biogenesis

**Figure 1:**
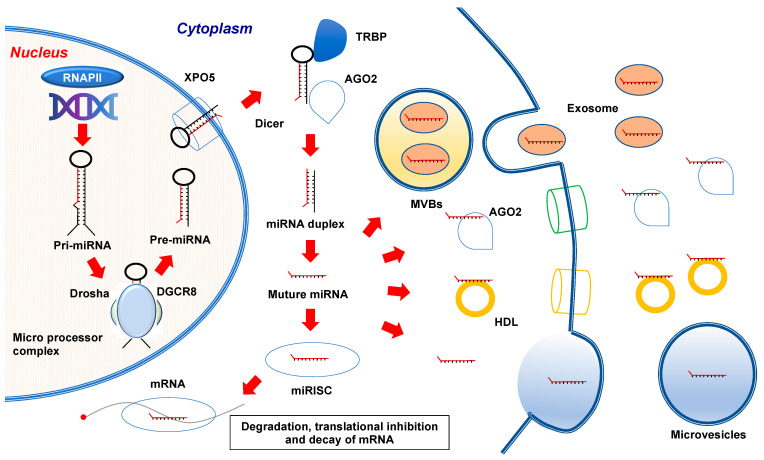
MicroRNA biogenesis. miRNAs are transcribed from the genome by RNA polymerase II (RNAP II) into primary transcripts (pri-miRNA) in the nucleus. Then, pri-miRNAs are cleaved into precursor miRNAs (pre-miRNAs) by Drosha to form a microprocessor complex with the DNA-binding protein DGCR8. Pre-miRNAs are exported to the cytoplasm by exportin-5 (XPO5), and then, Dicer, transactivating response RNA-binding protein (TRBP), and kinase R-activating protein (PACT) cleave pre-miRNAs in the cytoplasm. Finally, the guide miRNA generated by Dicer is loaded onto the RNA-induced silencing complex (RISC) consisting of Dicer, TRBP, PACT, and Argonaute 2 (AGO2), and subsequently binds to the 3′UTR of the target genes, inducing degradation or translational inhibition of the target mRNA. Mechanisms for release of circulating miRNAs: (i) Release through exosomes: first, endosomes are formed by invagination. Then, mature miRNAs are loaded into multivesicular bodies (MVBs). Finally, they are released from the cell membrane as exosomal miRNAs; (ii) Release through AGO2 and HDL: AGO2 and HDL bind to miRNAs and can be released into the extracellular space; (iii) Release through microvesicles: Microvesicles (MVs) are spontaneously generated from cells by direct budding and contain mature miRNAs.

miRNAs are primarily transcribed from the genome into primary transcripts (pri-miRNA) in the nucleus by RNA polymerase II (RNAP II) [9]. Then, the ribonuclease (RNase) III family enzyme Drosha, which forms a microprocessor complex with the DNA-binding protein DGCR8, processes pri-miRNAs into 70–80 nucleotide precursor miRNAs (pre-miRNAs) with a stem-loop structure [10]. Consequently, pre-miRNAs are exported to the cytoplasm by a Ran-GTP-dependent dsRNA-binding protein, exportin-5, and then pre-miRNAs are processed by Dicer (an RNase III-type endonuclease) into miRNA duplexes, which consist of a guide (miRNA) and a passenger (miRNA*) strand [11,12,13,14]. After cleavage by Dicer, the guide miRNA is retained by the RNA-induced silencing complex (RISC), which consists of Dicer, TRBP, PACT, Argonaute 2 (AGO2), and GW182/TNRC6 [13,15]. This miRNA and RISC complex detects the 3′UTR of target genes and promotes their downregulation [16,17]. Conversely, miRNA* undergoes degradation and exhibits no effect on gene regulation; however, several studies have reported that miRNA* is associated with the RISC complex and influences the expression of target mRNAs, similar to that of mature miRNA (Figure 1) [18].

## 3. Circulating miRNAs

Previously, miRNAs were thought to only function intracellularly because RNase degrades RNA in the extracellular environment. However, Valdi et al. [19] reported that miRNAs exist in culture medium and indicated that extracellular miRNAs might influence recipient cells. Subsequently, in 2008, Lawrie et al. [20] discovered a specific miRNA in the serum of patients with diffuse large B-cell lymphoma and reported, for the first time, a tumor-related miRNA that circulates in blood. These findings indicate the existence of extracellular miRNAs, which were subsequently recognized as circulating miRNAs. Circulating miRNAs were originally thought to translocate to the blood circulation from cells that have undergone apoptosis or necrosis, where they are detected as mature miRNAs in the plasma and serum [21,22]. Instead of such passive mechanisms of transfer from dead or injured cells, pathways in which cells actively secrete miRNAs have also been reported [21]. There is a pathway in which miRNAs are encapsulated in extracellular vesicles (EVs) consisting of cell membranes, which are secreted extracellularly. EVs are lipid bilayer-delimited particles that are released from human cells and are reported to have an important role in cell-to-cell communication [23]. In cancer, EVs are thought to influence cell proliferation, migration, invasion, and angiogenesis, and then induce cancer progression and metastasis [24,25,26].

EVs contain exosomes, the diameters of which range from 30 to 100 nm, and microvesicles, the diameters of which range from 100 to 1000 nm [27,28]. Previously, exosomes were recognized as cell waste products; however, several reports have indicated their importance in intercellular communication [29]. Exosomes are defined as vesicles secreted by cells derived from endosomes. The formation of exosomes begins with the formation of early endosomes by endocytosis. Subsequently, early endosomes mature to late endosomes, which contain intraluminal membrane vesicles (ILVs). Finally, multivesicular endosomes (MVBs) containing many ILVs fuse with the cell plasma membrane, and ILVs are released into the extracellular space as exosomes [30]. Via this process, miRNAs are sorted into exosomes and are called exosomal miRNAs. However, microvesicles (MVs) are spontaneously generated from cells by direct budding and contain mature miRNAs and AGO2-bound miRNAs with other molecules, including several types of proteins [31,32]. Data related to extracellular miRNAs are accumulating, and many of these miRNAs are reported to be released into the extracellular space bound to AGO2 and high-density lipoprotein (HDL) [33,34,35]. Because exogenous miRNAs are stabilized and effectively delivered to the recipient cells by these carriers, circulating miRNAs are considered to be useful as biomarkers (Figure 1) [36].

Once circulating miRNAs are released into extracellular space, for example, the blood, they can be transported to remote areas and affect recipient cells. It was reported that *miR-150*, which is specific for a leukocyte, was packaged into MVs and delivered to recipient cells [37]. Vickers et al. [38] revealed that HDL could transport miRNAs into recipient cells. They also showed that HDL-mediated *miRNA-223* transport repressed cholesterol uptake by binding to the scavenger receptor BI mRNA [38]. Thus, circulating *miRNAs* enter into recipient cells and interact with specific target genes. Biological effects are induced by circulating *miRNAs*. For example, Mittelbrunn et al. [39] reported that cellular communication via circulating *miRNAs* influences immunological synapses. In the study, they showed that exosomal *miRNAs* work as a mediator in directional transfer from T cells to antigen-presenting cells. Dendric cells (DCs) play an important role in regulating adaptive immunity. Montecalvo et al. [40] reported that DCs transferred cellular signals to neighbor DCs via exosomal *miRNAs*, and these *miRNAs* functioned as a means of communication and post-transcriptional regulation between DCs. Regarding other biological effects, Thomou et al. [41] revealed that adipose tissue releases circulating exosomal *miRNAs*, which regulate gene expression in distant liver tissue. These circulating *miRNAs*, which are derived from fat tissue, are thought to be a novel class of adipokines that regulate metabolism in distant tissue.

Regarding cancer-related circulating miRNAs, several studies demonstrated that circulating miRNAs are delivered to the target cells and induce the modulation of their mRNA targets. Tominaga et al. [42] reported cell-to-cell communication via *miR-181c*-containing EVs in breast cancer patients. To reveal the mechanisms of brain metastasis in breast cancer, they established brain metastasis breast cancer cell lines and investigated their interaction with the blood-brain barrier (BBB) in an in vitro model. Using those models, they found cancer cell-derived *miR-181c*-containing EVs could destroy the BBB and promote brain metastasis in breast cancer. The other report revealed that exosomal *miR-103* was secreted from hepatoma cell-downregulated multiple endothelial junction proteins. This study indicated that circulating miRNA influenced cell-to-cell junction, increasing vascular permeability and promoting metastasis of hepatoma [43].

Thus, circulating miRNAs have important roles in cellular communication and induce biological effects in several situations.

### 3.1. Detection of Circulating miRNAs

Though circulating miRNAs are considered to be possible markers for GIC, the detection of circulating miRNAs remains a challenge. In recent years, several studies have evaluated the expression of circulating miRNAs in cancer patients using polymerase chain reaction (PCR) [44,45]. Furthermore, new technologies, including microarray and next-generation sequencing, are capable of comprehensively detecting circulating miRNAs [46,47,48]. Current bioinformatic approaches, including various software and web-based analysis tools, have also improved the quality of analysis and made broad panel analysis possible. However, despite these technical improvements, several problems remain. Regarding sample quality control, the expression of circulating miRNAs can change depending on the sample collection method and sample type (full-blood or serum only). Specimen contamination can also cause problems with the accuracy of the results. Furthermore, unlike intracellular miRNAs, it is unknown which internal standard circulating miRNAs can be used as quantification controls, which is a barrier to the quantitative analysis of circulating miRNAs.

#### 3.1.1. Esophageal Cancer

Esophageal cancer is a malignant tumor and the sixth leading cause of cancer-associated deaths globally [1]. When detected early, the survival rate of patients with esophageal cancer is good; however, effective biomarkers that facilitate early diagnosis and therapy are lacking. miRNAs may serve as effective biomarkers for diagnosis, prognosis, and precision treatment in esophageal cancer.

The first study on the role of exosomal miRNA in esophageal cancer was conducted by Tanaka et al. [49]. They documented that the expression levels of exosomal *miR-21* were significantly higher in patients with esophageal squamous cell (ESCC) cancer than in those with benign diseases. Furthermore, they reported that exosomal *miR-21* expression was correlated with advanced tumor classification and lymph node and distant metastasis. Zhou et al. [50] also demonstrated that the differential expression of miRNAs in the plasma of esophageal squamous cell carcinoma patients may serve as a diagnostic biomarker. A six-miRNA signature of upregulated *miR-106a*, *miR-18a*, *miR-20b*, *miR-486-5p*, and *miR-584* and downregulated *miR-223-3p* in ESCC was identified, which could correctly discriminate esophageal squamous cell carcinoma patients from healthy controls. Hoshino et al. [51] assessed the role of *miR-1246* and *miR-106b* in the serum of patients with esophageal squamous cell carcinoma. The results revealed that *miR-1246* expression was significantly increased and *miR-106b* expression significantly decreased in each cohort. The area under the blood concentration–time curve (AUC) of the *miR-1246*/*miR-106b* ratio was 0.901 (sensitivity, 80.0%; specificity, 80.0%) and 0.903 (sensitivity, 82.1%; specificity, 82.3%), respectively, indicating a high diagnostic ability. The high *miR-1246*/*miR-106b* ratio group was associated with clinicopathological factors such as depth of invasion, progression, lymph node metastasis, and poor prognosis.

Meanwhile, Luo et al. [52] showed that *miR-339-5p* plays a direct role in radioresistance and prognosis. In their study, they found that *miR-339-5p* promotes radiosensitivity and higher serum *miR-339-5p* levels were positively associated with radiotherapy sensitivity and good survival. The proposed mechanism related to this observation is that *miR-339-5p* enhances radiosensitivity by targeting Cdc25A and is transcriptionally regulated by Runx3. To confirm this, correlations were observed between *miR-339-5p* levels and Cdc25A/Runx3 levels in tissue samples. The conclusion of this finding was that *miR-339-5p* can be used to predict the pathological response to preoperative radiotherapy in locally advanced esophageal squamous cell carcinoma, suggesting that it could be a promising noninvasive biomarker for facilitating personalized treatment. Recently, two reports were published concerning the usefulness of a new detection method of miRNA for diagnosis [53,54]. One is a novel absolute qPCR method that uses the AllGlo probe and exhibits a high application value in detecting miRNAs; the other is a combination of circulating miRNA and miRNA isoforms, referred to as isomiR, that is detected by next-generation sequencing. Using these methods, *miR-34a-5p*, *miR-148a-3p*, *miR-181a-5p*, *miR-30a-5p*, *miR-574-3p*, and *miR-205-5p* have been identified as potential biomarkers in the diagnosis and prognosis of esophageal cancer. Table 1 lists the circulating miRNAs linked to esophageal cancer in the order in which they were reported.

#### 3.1.2. Gastric Cancer

According to the most recent cancer statistics, gastric cancer is the third most common cancer in terms of incidence and mortality worldwide [1]. Although the therapeutic strategies related to gastric cancer have improved, including surgical resection, radiation, and chemotherapy, the prognosis for advanced gastric cancer remains poor [55]. Serum tumor biomarkers, including carcinoembryonic antigen (CEA), α-fetoprotein (AFP), and carbohydrate antigen 19-9 (CA19-9), are often used for gastrointestinal tumor detection, but their sensitivities and specificities are not sufficient for the early diagnosis of gastric cancer. Therefore, miRNAs as new diagnostic and prognostic biomarkers for gastric cancer are urgently required.

Several studies have shown the applicability of plasma or serum miRNAs as biomarkers to identify gastric cancer at an early stage. One of the earliest studies reported that the expression levels of *miR-221-3p*, *miR-376c-3p*, and *miR-744-5p* significantly changed in the serum of gastric cancer patients 5 years before the appearance of clinical symptoms [56]. Another study revealed that circulating *miR-196a* from plasma samples can serve as a potential biomarker for discriminating gastric cancer, and has a higher specificity and accuracy than the CEA method [57]. In addition, it was demonstrated that the expression levels of serum-circulating *miR-203* are closely associated with gastric cancer metastasis [58]. Furthermore, the levels of *miR-143-3p*, *miR-146a*, *miR-451a*, and *miR-501-3p* circulating in the serum appear to be a promising indicator for detecting whether gastric cancer patients will develop lymph node metastasis [59]. Conversely, a low *miR-101* plasma level is significantly associated with an advanced T factor, advanced disease stage, and peritoneal metastasis, and is a predictor of poor prognosis in gastric cancer patients [60].

In a study of exosomal miRNA, elevated serum exosomal levels of *miR-19b-3p* and *miR-106a-5p* were identified in gastric cancer patients, and their combined evaluation resulted in substantial improvements in gastric cancer diagnosis, superior to the clinically used serum biomarkers AFP and CA19-9 [61]. Furthermore, it was recently shown that exosomal *miR-10b-5p*, *miR-195-5p*, *miR-20a-3p*, and *miR-296-5p* levels are significantly elevated in gastric cancer patients as compared to healthy controls, and could be validated for their clinical diagnostic value [62]. In a recent report, the expression levels of exosomal *miR-217* and the combination of circulating *miR-21*, *miR-93*, *miR-106a*, and *miR-106b* were significantly higher in the serum of gastric cancer patients [63,64]. However, circulating *miRNA-22-3p* exhibited significantly lower expression in patients with precancerous lesions or gastric adenocarcinoma [65]. Gastric cancer patients with elevated exosomal *miR-423* and exosomal *miR-451* were associated with significantly shorter post-treatment survival [66,67].

Moreover, downregulation of exosomal *miR-23b* exhibited a significant association with liver metastasis, advanced TNM stage, tumor size, and tumor invasion, representing an independent predictor of risk for disease recurrence and poor survival outcome in gastric cancer [68]. Circulating *miR-21* is elevated in gastric cancer and has been found to be associated with numerous other cancer types [69]. A constructed miRNA-mRNA network showed that *CXCL5*, *CXCL9*, and *CXCL10* are target genes of *miR-588*, and high expression of *miR-588*, *CXCL5*, *CXCL9*, and *CXCL10* is associated with the prolonged survival in gastric cancer patients [70]. Table 2 shows the circulating miRNAs linked to gastric cancer in the order in which they were first reported.

#### 3.1.3. Colorectal Cancer

The overall incidence of colorectal cancer (CRC) is increasing, and the disease is the fourth leading cause of death worldwide [71]. Although early detection and current new treatment strategies have improved the prognosis of CRC, advanced-stage CRC remains difficult to cure. Several screening methods, including fecal occult blood barium enema and colonoscopy, are thought to be useful for detecting early-stage CRC; however, the wide use of these methods is limited because of their low efficacy, high cost, and invasiveness [72]. CEA, CA19-9, and carbohydrate antibody 72-4 (CA72-4) are used worldwide as noninvasive biomarkers, but these markers are not very sensitive nor specific for CRC [73]. For the above reasons, the clinical use of new noninvasive biomarkers is needed for early-stage detection and to improve the mortality of CRC.

Several recent studies have demonstrated the usefulness of circulating miRNAs as biomarkers for detecting early-stage CRC and evaluating prognosis. In one of the first reports, Chang et al. [74] evaluated the expression level of *miR-141* in the plasma samples of patients with CRC. They found that *miR-141* was significantly associated with stage IV CRC, and that the combined use of *miR-141* and CEA further improved the accuracy of CRC detection. *miR-21* is one of the earliest cancer-related miRNAs that influences tumor suppressor genes, affecting proliferation, apoptosis, and invasion in human malignancies [75]. Using qPCR, Tsukamoto et al. [76] found that exosomal *miR-21* is elevated and significantly associated with liver metastasis and TNM stage. They also analyzed the prognostic value of exosomal *miR-21* and indicated that exosomal *miR-21* is an independent prognostic factor for overall survival (OS) and disease-free survival (DFS) in stage II and III CRC patients and for OS in stage IV CRC patients.

Furthermore, *miR-21* has been reported as a biomarker for the early detection of CRC. Wikberg et al. [77] evaluated the expression of several types of miRNAs in the plasma of CRC patients; only the concentration of *miR-21* clearly increased during the 3 years prior to diagnosis. Thus, circulating miRNAs have been reported as both early CRC and advanced or metastatic CRC markers. Regarding the circulating miRNAs that are reported to be biomarkers for early-stage CRC, a higher expression of circulating *miR-338-5p* was reported to be a possible noninvasive diagnostic biomarker for CRC by Bilegsaikhan et al. [78]. They evaluated the expression level of circulating *miR-338-5p* in the serum samples of CRC patients and reported that circulating *miR-338-5p* in these patients was higher than in healthy controls. Furthermore, they found that the combination of *miR-338-5p* and CEA had the greatest diagnostic value [78].

Regarding the other diagnostic circulating miRNAs, our colleagues demonstrated that circulating *miR-199a-3p* is significantly higher in CRC patients than noncancer patients, and a high expression of circulating *miR-199a-3p* was associated with deep wall invasion using serum samples [79]. Furthermore, they reported that circulating *miR-103* and *miR-720* levels in serum are higher in CRC patients than in noncancer patients. In their study, *miR-103* expression was associated with histological differentiation grade and lymphatic invasion, while *miR-720* was associated with gender and lymph node metastasis [80]. Liu et al. [81] reported that circulating *miR-1290* and *miR-320d* are dysregulated in CRC patients. On the basis of the data from web-based datasets, they found that these two miRNAs were significantly downregulated, and they confirmed differential expression between CRC patient samples and healthy controls, which indicates that dysregulation of *miR-1290* and *miR-320d* could be a noninvasive biomarker for the early diagnosis of CRC [81].

By investigating clinical samples, Min et al. [82] compared circulating small extracellular vesicle (sEV) miRNAs and total plasma miRNAs as biomarkers of early CRC. They initially performed RNA sequencing using CRC clinical samples and analyzed The Cancer Genome Atlas (TCGA) dataset, which is an extensive web-based dataset of several cancer types. Then, they selected *let-7b-3p*, *miR-139-3p*, *miR-145-3p*, and *miR-150-3p* as candidates for further validation. In this study, the authors validated sEV-derived miRNAs that were enriched at a very early stage of CRC as compared to noncancerous controls and showed that the area under the ROC curve of sEV-derived miRNAs was higher than that of total plasma miRNAs, which indicates that sEV-derived miRNAs might be more accurate biomarkers for detecting CRC [82].

However, regarding the circulating miRNAs related to the prognosis of CRC, Yan et al. [83] revealed that five exosomal miRNAs (*miR-638*, *miR-5787*, *miR-8075*, *miR-6869-5p*, and *miR-548c-5p*) were downregulated and two exosomal miRNAs (*miR-486-5p* and *miR-3180-5p*) were upregulated in the sera of CRC patients. Among these miRNAs, the expression levels of exosomal *miR-638* were associated with an increased risk of liver metastasis and an advanced stage of CRC. Additionally, using bioinformatic methods, they mentioned that exosomal *miR-638* influences glucose metabolism and results in poor prognosis of CRC [83].

Takano et al. [84] also explored the relationship between circulating miRNA and metastasis in CRC. They focused on the interaction between monocytes and disseminated tumor cells (DTCs) and compared the expression levels of cancer-related mRNAs between monocytes and DTCs, both in CRC patients with metastasis and those without metastasis. On the basis of the above results, they analyzed mRNA expression and focused on circulating exosomal *miR-203* in CRC. In this study, a high expression of exosomal *miR-203* was significantly associated with poor OS and DFS. Moreover, when they evaluated the correlation between the exosomal *miR-203* expression and clinicopathological parameters of CRC patients, they found that a higher expression of exosomal *miR-203* was associated with a higher incidence of venous invasion, lymph node metastasis, distant metastasis, and perineal dissemination. Furthermore, through in vitro experiments, they revealed that exosomal *miR-203* from CRC cells could directly promote the differentiation of monocytes into M2 macrophages, forming a premetastatic niche and inducing metastasis and poor prognosis [84].

Another report by Fu et al. [85] revealed that circulating exosomal *miR-17-5p* and *miR-92a-3p* are significantly associated with the pathological stages and grades of CRC patients. Additionally, they tested several types of CRC cell lines with different gene mutations and metastatic abilities, which revealed that the expression levels of these miRNAs were associated with invasive and metastatic ability but not gene mutations (e.g., *KRAS, BRAF*, and *TP53*) [85].

The other circulating miRNAs that are reported as biomarkers of CRC are listed in Table 3 [86,87,88,89,90,91,92,93,94].

#### 3.1.4. Hepatocellular Cancer

Hepatocellular carcinoma (HCC) is one of the most common malignant tumors, ranking fourth in terms of cancer mortality worldwide [1]. Currently, numerous serum markers are used for HCC detection in clinical terms, such as AFP [95], but the sensitivity and specificity of AFP for the detection of HCC at an early stage are unsatisfactory [96,97]. Thus, noninvasive and effective diagnostic and prognostic biomarkers for early detection are urgently needed.

Many studies have demonstrated the potential of circulating miRNAs as biomarkers for HCC. Extracellular *miR-21* levels have been frequently documented as being significantly upregulated in cancer patients. In liver cancer, increased exosomal *miR-21* levels have significant diagnostic and prognostic properties due to their ability to discriminate cancer patients from chronic hepatitis B patients and healthy controls, and their positive correlation with advanced tumor stage [98]. Sugimachi et al. [99] reported that downregulation of serum exosomal *miR-718* is associated with advanced tumor stage, grade, and disease aggressiveness, and with a higher risk of tumor recurrence and poor survival.

Similarly, the downregulation of serum exosomal *miR-125b* has been associated with advanced tumor stage, grade, and disease aggressiveness, and with an increased risk of tumor recurrence and poor survival [100]. The circulating *miR-200* family members *miR-200a* and *miR-141* were shown to be significantly downregulated in the serum of HCC patients as compared to healthy controls and noncancerous liver cirrhosis patients. Both miRNAs are able to accurately discriminate patients with cirrhosis-associated HCC from healthy controls [101]. Cellular *miR-122* has been shown to be exclusively associated with the liver. Moreover, a higher expression of circulating *miR-122* was shown to be associated with poor OS at a confidence interval of 95% in patients with HBV-related carcinoma who had undergone radiofrequency ablation [102].

Sohn et al. [103] showed elevated *miR-222*, *miR-18a*, *miR-224*, and *miR-221* and decreased *miR-106b*, *miR-101*, and *miR-195* levels in the circulation of HCC patients and, thus, these miRNAs were proposed as potential novel serological biomarkers for the differential diagnosis of HCC patients from chronic hepatitis B and liver cirrhosis patients. Similarly, elevated *miR-519d*, *miR-595*, and *miR-939* levels were shown to be independent factors of HCC [104]. Other plasma-circulating miRNAs, such as *miR-224*, *miR-26a*, and *miR-29a,* were shown to be associated with HBV-related HCC patients and are therefore independent prognostic markers for poor disease-free survival [105,106]. Increased *miR-665* expression is correlated with a higher risk of disease metastasis and a worse survival outcome following hepatectomy [107]. More recently, similar observations were reported for *miR-93* and *miR-1247-3p* [108,109]. Exosomal *miR-93*, which directly targets *TP53INP1*, *TIMP2*, and *CDKN1A*, is significantly correlated with clinical features such as stage and tumor size [108]. Furthermore, a significant correlation between the survival rates of HCC patients and *miR-93* expression level has been reported. In hepatoblastoma, which occurs mostly in minors, *miR34a*, *miR-34b*, and *miR-34c* were reported as suitable noninvasive biomarkers for both diagnosis and prognosis [110]. A combination of AFP and *miR-122*, *miR-148a*, and *miR-1246* was shown to be highly accurate in discriminating HCC patients from healthy individuals [111]. Lin et al. [112] demonstrated that high levels of *miR-210-3p* in the serum of patients with HCC are correlated with higher microvessel density and thereby promote tumor angiogenesis by targeting *SMAD4* and *STAT6*. Serum exosomal *miR-638* in HCC patients is negatively associated with tumor size, vascular infiltration, and TNM stage [113]. In addition, the downregulation of serum exosomal *miR-638* is a predictor of poor prognosis in patients with HCC. Weis et al. [114] reported that their panel of three serum miRNAs (*miRNA-122-5p*, *miRNA-486-5p*, and *miRNA-142-3p*) distinguished HCC from cirrhosis, outperforming AFP. Numerous exosomal miRNAs have been reported to be potential novel biomarkers for the diagnosis and prognosis of HCC patients with/without a combination of traditional biomarkers [115,116,117]. Table 4 shows the circulating miRNAs linked to hepatocellular carcinoma cancer in the order in which they were reported.

#### 3.1.5. Pancreatic Cancer

Pancreatic cancer is one of the most prevalent malignant cancers, with a mean 5-year survival rate of 9%. Moreover, it is reported to be the seventh leading cause of cancer-related deaths globally [1]. Unfortunately, most patients are diagnosed at an inoperable advanced stage of the disease because early diagnostic and effective treatment strategies for pancreatic cancer are lacking; thus, there is an urgent need to explore diagnostic tools with a high sensitivity, specificity, and repeatability.

Circulating *miR-182* in plasma might be considered as a potential diagnostic and prognostic biomarker because its expression has been found to be significantly higher in pancreatic cancer patients, and this elevated expression is closely correlated with both shorter OS (*p* < 0.001) and DFS (*p* < 0.001) [118]. Furthermore, elevated *miR-10b* levels have been identified in plasma-derived exosomes of pancreatic cancer patients purified by the label-free and nondestructive sensing technique, and have been documented to discriminate pancreatic ductal adenocarcinoma from chronic pancreatitis patients or healthy controls [119]. Moreover, Madhavan et al. [120] showed that a combination of a panel of serum-exosomal miRNAs, including *miR-1246*, *miR-4644*, *miR-3976*, and *miR-4306*, and stem cell protein markers, including *CD104*, *EpCAM*, and *TSPAN8*, allows an extremely sensitive diagnosis of pancreatic cancer compared to the use of each marker individually. Furthermore, serum *miR-196* and *miR-200* plus CA19-9 improve the discriminative ability of the test as compared to CA19-9 alone [121].

Circulating exosomes from pancreatic ductal adenocarcinoma patients were found to have high levels of *miR-10b*, *miR-21*, *miR-30c*, and *miR-181a*, and this exomiR signature was demonstrated to distinguish pancreatic ductal adenocarcinoma patients from healthy controls or patients with chronic pancreatitis [122]. Elevated *miR-196* and *miR-1246* levels can discriminate patients with pancreatic ductal adenocarcinoma or intraductal papillary mucinous neoplasms from healthy individuals and chronic pancreatitis patients [123]. Our colleagues reported that an increase in *miR-155* through long-term exposure to gemcitabine induces the delivery of *miR-155* via exosome secretion and chemoresistance ability by facilitating antiapoptotic activity [124]. Furthermore, therapy targeted toward *miR-155* or exosome secretion effectively attenuates this chemoresistance, resulting in significant upregulation of *miR-21-5p* in the blood plasma of pancreatic cancer patients as compared to healthy individuals. In addition, increased expression of plasma-circulating *miR-21-5p* is significantly associated with the OS of pancreatic cancer patients [125].

Meanwhile, circulating *miR-100* can adequately discriminate pancreatic cancer patients from normal healthy controls with a high sensitivity and accuracy, and was found to be associated with pancreatic cancer progression [126]. The expression of *miR-221-3p* in plasma is correlated with distant metastasis and TNM stage, and the diagnostic efficacy of distant metastasis by *miR-221-3p* is better than that of CA19-9 [127]. Circulating exosomal *miR-222* and *miR-451a* are both strongly associated with tumor size, TNM stage, and worse OS [128,129]. Zou et al. [130] identified a six-miRNA (*let-7b-5p*, *miR-192-5p*, *miR-19a-3p*, *miR-19b-3p*, *miR-223-3p*, and *miR-25-3p*) panel in serum for the early diagnosis and prognosis of pancreatic cancer. Table 5 lists the circulating miRNAs linked to pancreatic cancer in the order in which they were reported.

## 4. Future Perspectives; Circulating miRNA-Based Therapy and miRNA Diagnostics

### 4.1. Circulating miRNA-Based Therapy

Several studies have focused on the possibility of using miRNAs in clinical applications, and various clinical studies are ongoing. Essentially, miRNA-based therapy is the replenishment of tumor-suppressive miRNAs or the injection of miRNA antagonists (anti-miRNAs), which targets oncological miRNAs-dependent tumors [131]. Anticancer therapy targeting miRNAs is thought to have minimal side-effects compared to protein-based drugs and DNA-based gene therapies. This is because miRNAs are antisense nucleotides. However, anti-miRNAs and miRNA mimics are ineffective without an adequate delivery system, because miRNAs are easily degraded by ribonucleases. In recent years, several types of delivery system have been developed, which is crucial. Our colleagues utilized super carbon apatite as a new drug delivery system targeting solid tumors, which can be seen as an miRNA-based therapy with a delivery system for GIC [132]. Using this system in a mouse xenograft model, Inoue et al. [133] reported that a complementary strand to *miR-29b-1-5p* had anticancer effects in KRAS-mutant CRC cells. A current study by Zhao et al. showed that *miR-139-5p* and surfaces decorated with epithelial cell adhesion molecule aptamer inhibited the proliferation, migration, and invasion of CRC cell lines in vivo and vitro [134]. In addition, Gokita et al. [135] reported the therapeutic potential of the lipid nanoparticle (LNP)-mediated delivery of *miR-634* in pancreatic cancer cells. Using a xenograft model, they confirmed a reduction in the xenograft tumor growth of pancreatic cancer cells.

Furthermore, several reports show the correlation between miRNAs and chemoresistance when using chemotherapy as a treatment for GIC. For example, a current report by Lin et al. [136] showed that exosomal *miR-500-3p* promoted cisplatin resistance in gastric cancer cells through the inhibition of *FBXW7*, which is a tumor suppressor gene. Additionally, the study revealed the upregulation of *miR-500-3p* in the plasma of gastric cancer patients with cisplatin resistance [136]. Liu et al. [137] revealed the correlation between exosomal *miR-128-3p* and chemosensitivity in CRC. They performed in vivo and in vitro studies using oxaliplatin-resistant CRC cell lines and revealed that exosomal *miR-128-3p* improved oxaliplatin resistance [137]. As regards the chemoresistance of HCC, Xiong et al. [138] developed a cyclodextrin-based star copolymer nanoparticle as a carrier material for doxorubicin (DOX) and *miR-122*. In an in vivo study, they confirmed that the preferentially released *miR-122* directly induced apoptosis and DOX accumulation in HCC [138]. Thus, several studies have revealed the potential of circulating miRNAs as anti-GIC reagent.

Regarding clinical trials related to miRNAs, miravirsen is a drug that affects hepatitis C virus (HCV) RNA by binding to two adjacent target sites in the 5′UTR region that are essential for its replication [139]. Consequently, miravirsen decreases the expression of HCV RNA in patients with HCV-induced cirrhosis and has completed a phase II clinical trial [140]. Regarding cancer therapies that target miRNAs, *miR-34*, in particular, is thought to be a potential oncological target and is now in a phase I trial for HCC and metastatic liver cancer (MRX34, NCT01829971, ClinicalTrials.gov, accessed on 19 May 2021). However, several problems remain to be addressed for therapeutic strategies targeting miRNAs, including identifying adequate and effective drug delivery systems and controlling the off- and on-target effects.

### 4.2. miRNA Diagnostics

As described above, a huge number of studies demonstrate the correlation between circulating miRNAs and GIC; however, circulating miRNAs have not yet been clinically applied as biomarkers. Because of their low specificity and sensitivity, it is thought to be difficult to detect a particular GIC using solitary miRNAs. Therefore, recent studies show that a panel of circulating miRNAs could better function as a diagnostic tool. Deng et al. [141] constructed a miRNA–mRNA network containing both pancreatic cancer and control samples from data extracted from the Gene Expression Omnibus database (https://www.ncbi.nlm.nih.gov/geo/, accessed on 19 May 2021; managed by the National Center for Biotechnology Information) and the Cancer Genome Atlas database (https://www.cancer.gov/about-nci/organization/ccg/research/structural-genomics/tcga, accessed on 19 May 2021; supervised by the National Cancer Institute’s Center for Cancer Genomics and the National Human Genome Research Institute). They identified a panel of eight miRNAs that would serve as early diagnostic biomarkers for PC patients. The ROC curves for each of the eight miRNAs demonstrated a strong separation between the tumor tissues and control group, with an AUC of 0.59–0.77. Furthermore, the panel of eight miRNAs could accurately distinguish all PC samples from the control samples [141]. Ibuki et al. [54] reported that a diagnostic panel that combines one mature miR and two isomiRs, both of which are useful for the diagnosis of esophageal cancer, is highly accurate for diagnosis after repeated validation.

Although a panel of circulating miRNAs remains a challenge, this promising biomarker can be adapted for use in GIC in future studies and clinical trials.

## 5. Conclusions

There remain challenges related to the clinical application of miRNA-based therapy for GIC. This is because the safety of microRNAs is not guaranteed and a drug delivery system has not yet been established. However, as biomarkers for cancer, miRNAs are useful tools for detecting early-stage cancers and the progression of tumors and metastasis, especially since miRNAs in body fluids and extracellular vesicles are stable. Following the development of more precise and convenient detection methods, circulating miRNAs may be used as oncological markers in a similar way to conventional markers.

Furthermore, current studies focused on defining panels of circulating miRNAs in the blood of cancer patients will provide new diagnostic strategies and precision medicine in the near future.

## Figures and Tables

**Table 1:** Esophageal cancer.

miRNA	Sample	Regulation	Significance	Sensitivity and Specificity in Diagnosis	Correlation between miRNA Expression and Clinicopathological Parameter	References
*miR-21*	serum exosome	↑	Diagnosis and Prognosis	-	differentiation, lymph node metastasis, distant metastasis, pathological stage	[49]
*miR-106a*, *miR-18a*, *miR-20b*, *miR-486-5p*, *miR-584*	plasma	↑	Diagnosis	85.7%, 95.8%	-	[50]
*miR-223-3p*	plasma	↓	Diagnosis	85.7%, 95.8%	-	[50]
*miR-339-5p*	serum	↓	Treatment response and Prognosis	-	pathological stage	[52]
*miR-34a-5p*	plasma	↑	Diagnosis	85.45%, 84.71%	-	[53]
*miR-148a-3p*, *miR-181a-5p*	plasma	↓	Diagnosis	85.45%, 84.71%	-	[53]
*miR-1246*	serum	↑	Diagnosis and Prognosis	71.3%, 70.6%	depth of invasion, lymph node metastasis	[51]
*miR-106b*	serum	↓	Diagnosis and Prognosis	74.3%, 73.5%	depth of invasion, lymph node metastasis	[51]
*miR-30a-5p*, *miR-574-3p*, *miR-205-5p*	serum	↑	Diagnosis	88.9%, 72.3%	-	[54]

↑; Upregulated, ↓; Downregulated.

**Table 2:** Gastric cancer.

miRNA	Sample	Regulation	Significance	Sensitivity and Specificity in Diagnosis	Correlation between miRNA Expression and Clinicopathological Parameter	References
*miR-221*, *miR-744*, *miR-376c*	serum	↑	Diagnosis	82.4%, 58.8%	differentiation	[56]
*miR-196a*	Plasma	↑	Diagnosis and Prognosis	69.5%, 97.6%	distant metastasis, pathological stage	[57]
*miR-203*	serum	↓	Prognosis	-	depth of invasion, lymph node metastasis, distant metastasis	[58]
*miR-143-3p*, *miR-146a*, *miR-451a*, *miR-501-3p*	serum	↓	Prognosis	-	lymph node metastasis	[59]
*miR-101*	plasma	↓	Prognosis	-	depth of invasion, pathological stage, peritoneal metastasis	[60]
*miR-19b-3p*, *miR-106a-5p*	serum exosome	↑	Diagnosis and Prognosis	95%, 90%	clinical stage, lymph node metastasis	[61]
*miR-10b-5p*, *miR-195-5p*, *miR-20a-3p*, *miR-296-5p*	serum exosome	↑	Diagnosis	-	-	[62]
*miR-106a*, *miR-106b*, *miR-21*, *miR-93*	plasma	↑	Diagnosis	84.8%, 79.2%	-	[63]
*miR-22-3p*	plasma	↓	Diagnosis	91.70%, 65.40%	-	[65]
*miR-217*	serum exosome	↑	Diagnosis	81.3%, 83.2%	pathological stage	[64]
*miR-423-5p*	serum exosome	↑	Diagnosis and Prognosis	81.0%, 57.5%	lymph node metastasis	[66]
*miR-451*	serum exosome	↑	Prognosis	-	-	[67]
*miR-23b*	serum exosome	↓	Prognosis	-	tumor size, depth of invasion, liver metastasis, pathological stage	[68]
*miR-21*	plasma	↑	Diagnosis	86.7%, 72.2%	-	[69]
*miR-588*	plasma	↓	Diagnosis and Prognosis	-	-	[70]

↑; Upregulated, ↓; Downregulated.

**Table 3:** Colorectal cancer.

miRNA	Sample	Regulation	Significance	Sensitivity and Specificity in Diagnosis	Correlation between miRNA Expression and Clinicopathological Parameter	References
*miR-141*	plasma	↑	Prognosis	77.1%, 89.7%	pathological stage	[74]
*miR-199a-3p*	serum	↑	Diagnosis	47.6%, 75.0%	depth of invasion	[79]
*miR-19a*	serum exosome	↑	Prognosis	-	depth of invasion, lymph node metastasis, liver metastasis, pathological stage	[86]
*miR-103* and *miR-720*	serum	↑	Diagnosis	55.9%, 75%	differentiation, lymph node metastasis	[80]
*miR-4772-3p*	serum exosome	↓	Prognosis	78.6%, 77.1%	-	[87]
*miR-21*	plasma exosome	↑	Prognosis	-	liver metastasis, pathological stage	[76]
*miR-638*	serum exosome	↓	Prognosis	-	liver metastasis, pathological stage	[83]
*miR-203*	serum exosome	↑	Prognosis	-	distant metastasis	[84]
*miR-6803-5p*	serum exosome	↑	Prognosis	-	lymph node metastasis, liver metastasis, pathological stage	[88]
*miR-17-5p* and *miR-92-3*	serum exosome	↑	Prognosis	-	pathological stage	[85]
*miR-21*	plasma	↑	Diagnosis	90%, 81%	-	[77]
*miR-27a* and *miR-130a*	plasma exosome	↑	Diagnosis and Prognosis	85.2%, 90.9%	-	[89]
*miR-338-5p*	serum	↑	Diagnosis	85%, 88.8%	-	[78]
*miR-200c* and *miR-141*	serum exosome	↑	Prognosis	-	-	[90]
*miR-548c-5p*	serum exosome	↓	Prognosis	-	venous invasion, distant metastasis, pathological stage	[91]
*miR-17-5p*, *miR-18a-5p*, *miR-181a-5p* and *miR-18b-5p*	plasma exosome	↑	Diagnosis	76.9%, 86.7%	-	[92]
*miR-1290* and *miR-320d*	plasma	↓	Diagnosis	90.9%, 93.3%	-	[81]
*miR-144-3p*, *miR-425-5p* and *miR-1260b*	plasma	↓	Diagnosis	93.8%, 91.3%	-	[93]
*miR-497*	serum	↓	Diagnosis and Prognosis	80.9%, 81.4%	differentiation, lymph node metastasis, pathological stage	[94]
*miR-139-3p* and *miR-145-3p*	plasma exosome	↓	Diagnosis	-	-	[82]
*miR-150-3p* and *let-7b-3p*	plasma exosome	↑	Diagnosis	-	-	[82]

↑; Upregulated, ↓; Downregulated.

**Table 4:** Hepatocellular cancer.

miRNA	Sample	Regulation	Significance	Sensitivity and Specificity in Diagnosis	Correlation between miRNA Expression and Clinicopathological Parameter	References
*miR-21*	serum exosome	↑	Diagnosis and Prognosis	-	cirrhosis, pathological stage	[98]
*miR-718*	serum exosome	↓	Prognosis	-	differentiation, tumor size, tumor number	[99]
*miR-141*, *miR-200a*	serum	↓	Diagnosis	79%, 72%	-	[101]
*miR-122*	plasma	↑	Prognosis	-	-	[102]
*miR-222*, *miR-18a*, *miR-224*, *miR-221*	serum exosome	↑	Diagnosis	-	-	[103]
*miR-106b*, *miR-101*, *miR-122*, *miR-195*	serum exosome	↓	Diagnosis	-	-	[103]
*miR-519d*, *miR-595*, *miR-939*	serum	↑	Diagnosis	-	-	[104]
*miR-224*	plasma	↑	Diagnosis	93.1%, 80.0%	-	[105]
*miR-26a*, *miR-29a*	plasma	↓	Prognosis	-	tumor size	[106]
*miR-665*	serum exosome	↑	Prognosis	-	tumor size, local invasion, pathological stage	[107]
*miR-34a*, *miR-34b*, *miR-34c*	serum exosome	↓	Diagnosis	-	-	[110]
*miR-125b*	serum exosome	↓	Prognosis	-	tumor number, encapsulation, pathological stage	[100]
*miR-122*, *miR-148a*, *miR-1246*	serum exosome	↑	Diagnosis	87.0%, 90.0%	-	[111]
*miR-210-3p*	serum	↑	Diagnosis	-	-	[112]
*miR-93*	serum exosome	↑	Diagnosis and Prognosis	-	tumor size, pathological stage	[108]
*miR-1247-3p*	serum exosome	↑	Prognosis	-	lung metastasis	[109]
*miR-638*	serum exosome	↓	Prognosis	-	tumor size, vascular invasion, pathological stage	[113]
*miR-122-5p*, *miR-486-5p*, *miR-142-3p*	serum	↑	Diagnosis	80%, 95%	-	[114]
*miR-122a*	serum	↓	Diagnosis	-	-	[115]
*miR-548-a-3p*	serum	↓	Diagnosis and Prognosis	92%, 69.2%	-	[116]
*miR-1246*	serum	↑	Prognosis	-	differentiation, lymph node metastasis, portal vein invasion	[117]

↑; Upregulated, ↓; Downregulated.

**Table 5:** Pancreatic cancer.

miRNA	Sample	Regulation	Significance	Sensitivity and Specificity in Diagnosis	Correlation between miRNA Expression and Clinicopathological Parameter	References
*miR-182*	plasma	↑	Diagnosis and Prognosis	64.1%, 82.6%	lymph node metastasis, pathological stage	[118]
*miR-10b*	serum exosome	↑	Diagnosis	-	-	[119]
*miR-1246*, *miR-4644*, *miR-3976*, *miR-4306*	serum exosome	↑	Diagnosis	100%, 80%	-	[120]
*miR-196*, *miR-200*	serum	↑	Diagnosis	94%, 82%	-	[121]
*miR-10b*, *miR-21*, *miR-30c*, *miR-181a*	plasma	↑	Diagnosis	100%, 100%	-	[122]
*miR-196*, *miR-1246*	serum exosome	↑	Diagnosis	-	-	[123]
*miR-155*	serum exosome	↑	Treatment response	-	-	[124]
*miR-21-5p*	plasma	↑	Diagnosis and Prognosis	85%, 100%	-	[125]
*miR-100*	serum	↓	Diagnosis and Prognosis	-	-	[126]
*miR-221-3p*	plasma	↑	Diagnosis and Prognosis	76.3%, 63.6%	distant metastasis, pathological stage	[127]
*miR-222*	serum exosome	↑	Prognosis	-	tumor size, pathological stage	[128]
*miR-451a*	serum exosome	↑	Prognosis	-	tumor size, pathological stage	[129]
*miR-19a-3p*, *miR-19b-3p*, *miR-25-3p*, *miR-192-5p*, *miR-223-3p*	serum	↑	Diagnosis and Prognosis	95.3%, 76.7%	-	[130]

↑; Upregulated, ↓; Downregulated.

## Data Availability

No new data were created or analyzed in this study. Data sharing is not applicable to this article.
